# Uniaxial Tensile Behavior, Flexural Properties, Empirical Calculation and Microstructure of Multi-Scale Fiber Reinforced Cement-Based Material at Elevated Temperature

**DOI:** 10.3390/ma14081827

**Published:** 2021-04-07

**Authors:** Li Li, Mehran Khan, Chengying Bai, Ke Shi

**Affiliations:** 1 Key Laboratory of Agricultural Soil and Water Engineering in Arid and Semiarid Areas of Ministry of Education, College of Water Resources and Architectural Engineering, Northwest A&F University, Yangling 712100, China; 2 State Key Laboratory of Green Building Materials, China Building Materials Academy, Beijing 100024, China; 3 School of Civil Engineering, Dalian University of Technology, Dalian 116024, China; drmehrankhan@outlook.com; 4 Key Laboratory of Superlight Materials and Surface Technology, Ministry of Education, College of Materials Science and Chemical Engineering, Harbin Engineering University, Harbin 150001, China; chengyingbai@163.com; 5 School of Civil Engineering and Architecture, Zhengzhou University of Aeronautics, Zhengzhou 450046, China; shike@zua.edu.cn

**Keywords:** whiskers, fiber reinforced composite, hybrid fibers, high temperature, uniaxial tensile, bending strength

## Abstract

Fire is one of the most unfavorable conditions that cement-based composites can face during their service lives. The uniaxial tensile and flexural tensile properties of the steel-polyvinyl alcohol fiber-calcium carbonate whisker (CW) multi-scale fiber reinforced cement matrix composites (MSFRCs) under high temperatures are studied, including strength, deformation capacity, energy dissipation capacity, and its ability to be assessed through the empirical calculation method. The study showed that with the increase of the treatment temperature, the MSFRC residual bending strength, bending toughness, and tensile strength decreased overall, but the decline was slow at 600 °C. The peak flexural deflection and peak tensile strain of MSFRC first reduced and then increased with the increase of the temperature. As the temperature increased, the nominal stiffness of MSFRC bending and straight gradually reduced, and the rate of decline was faster than that of its strength. However, the uniaxial tensile properties were more sensitive to the temperature and degraded more rapidly. A quantitative relationship was established between MSFRC residual bending, tensile strength, and temperature. A comparison with existing research results shows that MSFRC has achieved an ideal effect of high temperature resistance. The multi-scale hybrid fiber system significantly alleviates the deterioration of cement-based composite’s mechanical properties under high temperatures. With the help of an optical microscope and scanning electron microscope (SEM), the high temperature influence mechanism on the uniaxial tensile and flexural properties of MSFRC was revealed.

## 1. Introduction

The inclusion of fiber effectively controls the cracking and improves cement-based composites’ toughness [1,2,3,4]. Fiber reinforced concrete has been widely used in engineering applications worldwide. For example, cladding fiber reinforced concrete panels were used in Foundation Louis Vuitton, France, and a fiber reinforced concrete roof was used in Shawnessy Light Rail Train Station, Canada [5]. However, a single scale’s macroscopic or microscopic fiber is difficult to fit alongside the multi-scale structural characteristics and multi-stage cracking process of cement-based materials. So, fibers’ strengthening and toughening effect on cement-based composites is limited [6,7]. In this paper, a micron CaCO_3_ whisker (CW) was introduced into a hybrid system of steel fiber and polyvinyl alcohol (PVA) fiber to develop multi-scale fiber reinforced cement matrix composites (MSFRCs). This MSFRC can achieve multi-level and multi-scale strengthening of cement matrix composites and improve the flexural tensile strength, flexural tensile strength, and toughness of cement matrix composites [8,9,10,11,12,13,14,15]. Other studies have shown that CW could be mixed with macroscopic fibers to improve composite mechanical properties. These composites include oil well cement [16], engineered cement-based composites (ECC) [17], foamed concrete [18], and normal concrete [19], which has a broad application prospect. For example, it can be used as a roof, cladding panel, bridge deck, and retrofit material. Previous studies showed that the addition of fibers is an effective way to improve the strength of composites [20,21,22]. The application of hybrid fibers in cement matrix composites in the construction industry has gained considerable developments during past the few decades [23,24,25,26,27]. Steel fibers showed higher thermal conductivity and thermal stability with crack resistance phenomena against thermal stress, even under high temperatures because of effective heat transfer phenomena. The steel fiber provides the bridging effect in fiber reinforced concrete over a large temperature range and does not melt at temperatures less than 1300 °C [9,28,29,30]. Previous studies showed that the addition of PVA fiber had considerably enhanced the mechanical properties and toughness of composites and performed as a compatible reinforcing fiber for cementitious composites [10,12,14]. In addition, the calcium carbonate whisker (CW) as a micro-fiber performed well at the micro-level and decomposed at 800–900 °C in composites [7,31,32,33,34,35]. Furthermore, the two or more hybrid fibers varying in type, tensile strength, elastic modulus, length, and shapes can provide better mechanical parameters and crack resistance at multi-stages in cement based composites.

Fire is a frequently occurring disaster, which seriously threatens the safety of people’s lives and property. After a fire, the performance analysis and evaluation of exposed buildings are the basis of reliable reinforcement and recovery of bearing capacity and service functions of facilities. It is undoubtedly essential to evaluate and analyze the main structural materials performance after fire and high temperatures. Cement-based materials undergo drastic and complex physical and chemical changes at high temperatures, leading to the deterioration of cement-based materials properties and even complete loss of bearing capacity. CW will undergo a phase transition at high temperatures [31,34,35,36]. Macroscopic fibers also undergo significant physical and chemical changes, leading to degenerations in the mechanical properties of the new MSFRC under high temperatures. For micro and meso cracks, one third of the fiber length can be taken as the allowable crack limit with an assumption of one third of the fiber length as the development length. For meso and macro cracks, the crack width can be taken as the fiber length minus two times the development length. However, the research on the high temperature performance of CW and macroscopic fiber hybrid reinforced cement-based materials is still relatively scarce [9]. Therefore, in this study, the new MSFRC with CW and steel-PVA hybrid fiber is evaluated for better performance under high temperatures.

The hybrid fiber reinforced composites are designed in a mode that provides the synergetic effect with maximum advantage taken from each type of fiber under mechanical loading, ultimately offering better mechanical characteristics. The aim of the current study is to evaluate the new MSFRC under high temperatures as well as to propose a new computation model high temperature effect for research purposes. The addition of multi-scale fibers (micro, meso, and macro fiber) will contribute at different levels (micro, meso, and macro levels) in composites and their combined effect will provide resistance against cracking at multi-stages. To promote the fire resistance engineering application of new MSFRC and steel-PVA hybrid fiber reinforced cement matrix composites, the direct uniaxial and bending tensile behavior of MSFRC at room temperature (25 °C), 200 °C, 400 °C, and 600 °C was studied. An empirical calculation method for the variation of tensile strength and bending strength with various temperatures was established. The microstructure of MSFRC was analyzed by combining it with morphology observations at different scales.

## 2. Materials and Mix Proportion 

### 2.1. Properties of Raw Materials

(1) Ordinary Portland cement: P.O42.5R type, the chemical composition is shown in Table 1, performance index and origin are shown in Table 1.
(2) Quartz sand: three kinds of Dalian local quartz sand were selected and obtained by gradation adjustment. The performance indexes and origin are shown in Table 1.
(3) Polycarboxylic acid superplasticizer: ASTM C494F type, with a water reduction efficiency of 24.1%, Sika (China) Co., Ltd., Suzhou, China.
(4) CW and Fiber: The basic properties and origin of CW, PVA fiber, and steel fiber are shown in Table 1.

### 2.2. Mix Proportion

The water-cement ratio of 0.3 and the sand-cement ratio of 0.5 were adopted. The water-reducing agent dosage was adjusted to ensure each fresh mortar’s consistent fluidity. The volume contents of steel fiber, PVA fiber, and CW were 1.25%, 0.5%, and 3%, respectively, and the mass content is 117, 6.45, and 85.8 kg/m^3^, respectively. On the other hand, the advantage for mixing hybrid fibers in cement matrix composites will give rise to complementary benefits in term of better direct uniaxial and bending tensile properties for the reason that various fibers will contribute individually and play their bridging part at their particular micro, meso and macro scale under elevated temperature.

**Figure 1:**
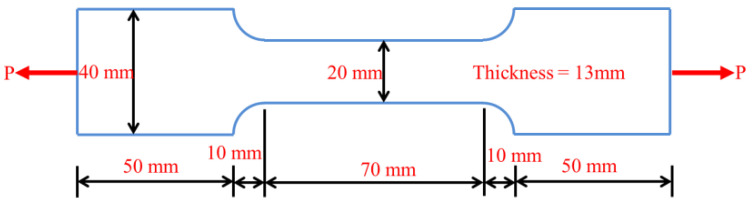
Shape and size of uniaxial tensile specimen.

The shape and size of uniaxial tensile specimen are presented in Figure 1. It has a variable cross section with a total length of 190 mm. The cross section of both the top and bottom part of the specimen is 40 mm × 13 mm with a length of 50 mm. The cross section of the middle measuring part is 20 mm × 13 mm with a length of 70 mm, and the variable cross-sectional segment has a length of 10 mm, which is curve-designed with a curvature radius of 14.41 mm. In this way, the cross-sectional area of the measuring part is smaller than that of the variable cross-sectional part, avoiding the breaking of the expected gauge length [37]. 

## 3. Experimental Procedure

### 3.1. High Temperature Treatment Procedure 

The specimen was molded and cured to 28 days of age for high temperature treatment. Pre-drying before high temperature treatment was carried out to reduce the specimen’s moisture content as much as possible. A pre-drying method involved 60 °C oven drying for 48 h. The heating rate of high temperature treatment is 3 °C/min. After reaching the target temperature (200, 400, and 600 °C, respectively), the temperature was kept constant for 2 h and then cooled to room temperature with the furnace.

### 3.2. Uniaxial Tensile and Four Point Bending Test Methods

**Figure 2:**
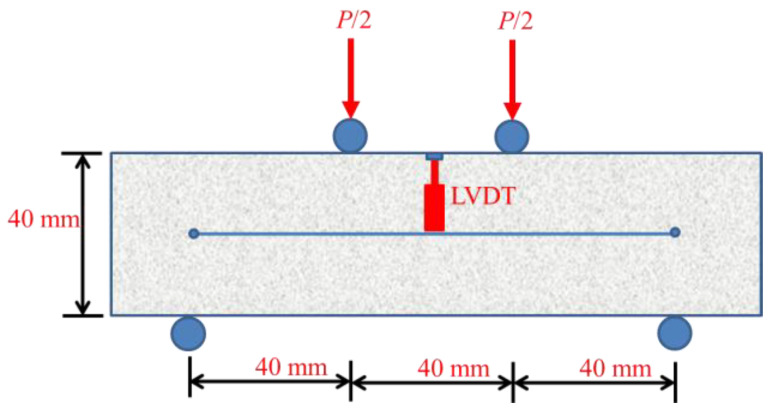
Experimental setup of four-point flexural loading.

For uniaxial tensile performance, self-designed dog-bone thin plate specimens were adopted. The size and loading mode of samples are shown in Figure 1. A uniaxial tensile test was carried out using MTS hydraulic servo testing machine (MTS Industrial Systems (China) Co., Ltd., Shenzhen, China), and the loading rate was 0.1 mm/min. The gauge length was 50 mm in the middle of the dog-bone specimen, and the test was terminated when the strain was no less than 2%. In the test, the MTS testing machine’s load and displacement sensors were used to collect the full uniaxial tensile stress-strain curves of the specimens for analysis. A total of four uniaxial tensile specimens were tested for each mixture at each temperature. As shown in Figure 2, prism specimens with a size of 40 mm × 40 mm × 160 mm were used to do the four-point bending test after high temperature treatment. Displacement control was adopted with loading rate of 0.05 mm/min. We used load sensors (Zhongcheng Sensor Co., Ltd., Bengbu, China) to collect load change and a Linear Variable Differential Transformer (LVDT) displacement sensor to acquire the beam span deflection change. A total of four bending specimens were tested for each mixture at each temperature.

### 3.3. Multi-Scale Morphological Observation

After the mechanical tests of MSFRC, a sample with a diameter of about 10 mm was made. Multi-scale observation of microstructure and micromorphology was carried out with an optical microscope (OM, Carl Zeiss AG, Oberkochen, Germany) and scanning electron microscope (SEM, Thermo Fisher Scientific, Hillsboro, U.S.) to analyze the multi-scale fiber system’s structural evolution mechanism at different scales.

## 4. Results and Discussion

### 4.1. Bending Performance

(1) Flexural strength

**Figure 3:**
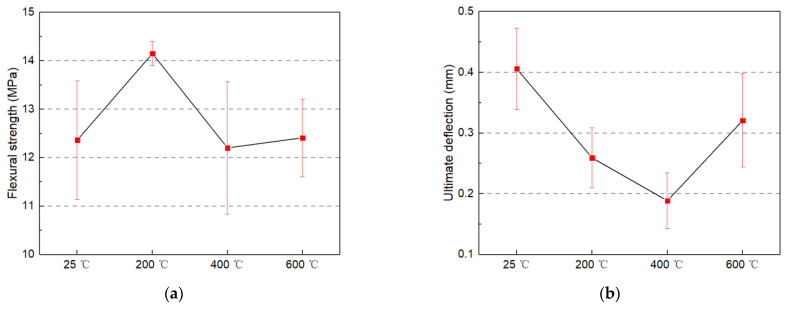
Bending performance: (**a**) Flexural strength; and (**b**) Ultimate deflection of heated multi-scale fiber reinforced cement matrix composite (MSFRC).

Figure 3a shows the influence of temperature on residual flexural strength of MSFRC after high temperature treatment. When the temperature is between room temperature and 600 °C, the residual bending strength decreases slowly with the increased temperature (by about 30%). At 200 °C, there was an increase in bending strength. Specifically, when the temperature is between 200 and 600 °C, the maximum residual bending strength can reach 114.5% of the specimen without high temperature treatment. The reason is that the hot steam environment in the mortar during high temperature treatment accelerates the continuous hydration of un-hydrated cement particles and fly ash particles, thus alleviating the deterioration of bending and tensile strength caused by the decomposition of hydration products [38,39]. Because CW dispersion in the matrix results in a “nucleation” effect, similar to that of cement hydration [7,40]. In other words, the newly produced hydration products under the action of high temperature steam are easy to adhere to and gather on the surface of CW, which strengthens the crack inhibition effect of CW [34]. The improvement of the CW effect further enhances the crack inhibition and reinforcement of steel fiber and PVA fiber, thereby improving the flexural strength of MSFRC.

The excellent fire resistance of steel fiber and CW below 600 °C is also a prerequisite to ensure the regular operation of MSFRC and play its role of crack resistance and enhancement. The PVA-steel hybrid fiber ultra-high toughness cement-based composite designed by Li et al. [39] presented good deformation and energy dissipation capacity at room temperature. But its residual flexural strength at 400 °C is only 55.6% of that at room temperature, while the MSFRC in this paper is 114.5%. The reason for this difference is that the specimen of Li et al. [39] added a high content of PVA fiber, but the PVA fiber had been melted at high temperatures before 400 °C and could not usually play its cracking inhibition role. In contrast, this paper’s specimen adopts a high steel fiber and CW content, ensuring that it can still work, usually at 600 °C. Hence, the crack resistance is enhanced without severe bending and tensile strength degradation.

(2) Flexural load-deflection curves

**Figure 4:**
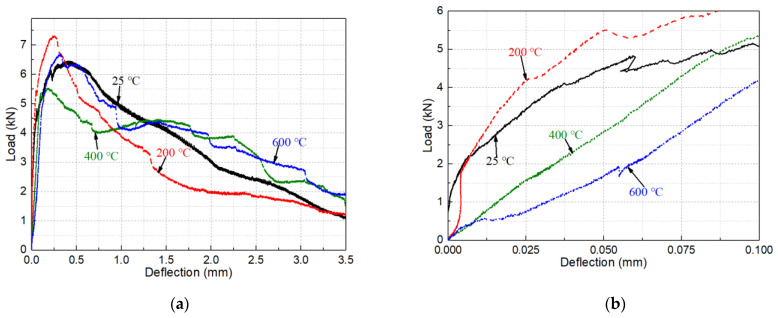
Effect of temperature on the load-deflection curve of heated MSFRC at (**a**) 0–3.5 mm and (**b**) 0–0.1 mm.

The load-deflection curve is an essential tool for analyzing cement-based composites’ flexural performance. The full deflection load-deflection of flexural test can analyze many indexes such as nominal stiffness (slope of the curve), peak load, peak deflection (deflection corresponding to peak load), flexural toughness (area under the curve), and so on [41]. Figure 4 shows typical load-deflection curves of MSFRC treated at different temperatures from room temperature to 600 °C. The load-deflection curve of MSFRC after the high temperature is roughly the same as that at room temperature, showing ductile failure, as shown in Figure 4a. As shown in Figure 4b of 0–0.1 mm deflection range of load-deflection curve. While the initial curve fluctuates, it can still be found that at 200 °C, the flexural rigidity was not significantly reduced and even appeared to show a certain degree of increase. This is because the matrix’s strength and stiffness are improved due to the evaporation of free water, and the PVA fiber is hardened by heat, improving the nominal stiffness of the MSFRC specimen. With the temperature increasing, the nominal stiffness of 400 and 600 °C specimens decreased significantly. Specifically, the nominal stiffness of 400 and 600 °C specimens decreased by about 20% and 50%, respectively, compared with those at room temperature. This is caused by PVA fiber melting and matrix deterioration at high temperatures. From room temperature to 400 °C, the peak deflection was decreased gradually, as shown in Figure 3b and Figure 4a. The reason for this change is that the PVA fiber with strong deformability will be hardened by heat at 200 °C and melted by heat at 400 °C, with both leading to the decrease of the deformability. Also, free water evaporation in the matrix below 200 °C decreases deformation capacity. As the temperature continued to rise to 600 °C, the peak load-deflection was increased. The main reasons are the deterioration of the matrix at high temperature and the softening of the steel fiber, both of which will decrease the specimen’s stiffness and increase the deformation.

As shown in Figure 4a, the MSFRC load-deflection curve becomes steeper. The area under the curve decreases as the room temperature increases to 200 °C, caused by the matrix and PVA fiber’s reduced deformation capacity. As the temperature continued to rise to 400 and 600 °C, the peak load decreases somewhat, but the curve’s descending stage becomes more gentle and full. Under the high temperature of 400–600 °C, the PVA fiber has completely melted and cannot play its role, so the peak load decreases to different degrees. There is high temperature water vapor in cement-based materials during high temperature treatment, accelerating the continuous hydration of unhydrated cement particles and fly ash. CW presents a similar “crystal nucleus” effect, as the newly generated hydration products are accessible to aggregate and adhere to the CW surface. Around 400 °C, CW changes from the aragonite type to the calcite type. The bonding force between the cement stone and calcite type CaCO_3_ is more vital than that of aragonite type [35,42]. As a result, CW’s “crystal nucleus” effect improved the micro-crack control and energy dissipation capacity. Finally, under the combined action of internal curing and CW, the defects formed by high temperature are filled to a certain extent. The interface between steel fiber and matrix is strengthened, which alleviates the bending property degradation of MSFRC caused by high temperatures.

### 4.2. Uniaxial Tensile Properties

(1) Uniaxial tensile strength

**Figure 5:**
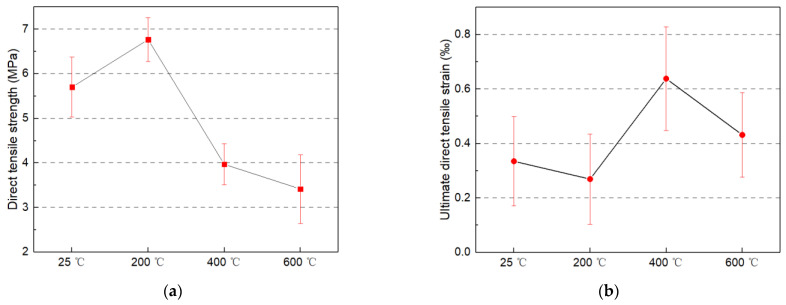
Uniaxial tensile behavior: (**a**) Tensile strength; and (**b**) Tensile strain of heated MSFRC.

The variation trend of MSFRC residual uniaxial tensile strength with temperature after high temperature treatment is consistent with the flexural strength. It increased somewhat after high temperature treatment at 200 °C, and then slowly decreased, as shown in Figure 5a. Compared with the flexural tensile strength, the uniaxial tensile strength degenerated faster with the temperature increase. For example, compared with the specimens without high temperature treatment, after 400 °C high temperature treatment, the uniaxial tensile strength was decreased by 30.4%. In contrast, the flexural strength was only reduced by 1.3%, which is consistent with the research results of steel-PP hybrid fiber reinforced concrete at high temperatures in the literature [43]. The reason for this is that after the high temperature of 400 °C, in addition to the cracks and pores caused by the decomposition of cement hydration products and thermal expansion, the PVA fibers have entirely melted and left more pores in MSFRC. Under uniaxial tensile load, the whole section of the specimen is under the action of tensile stress, so it is more sensitive to defects. Meanwhile under the action of flexural load, only part of the section of the specimen is under the action of tensile stress, so it is not sensitive to the melting of PVA and the increase of porosity. In addition, the size of the flexural specimen used in this paper is more significant than that of the uniaxial tensile specimen, so the effect of high temperature in the flexural specimen is more significant than that in the uniaxial tensile specimen. With the interaction of newly generated hydration products and CW, the action effect of steel fiber is strengthened, so flexural strength reduction is not significant.

The uniaxial tensile strength of MSFRC at 600 °C decreased compared with that at 400 °C, but the decrease rate slowed down obviously. As mentioned above, this is because the PVA fiber decomposes at 400 °C, so the uniaxial tensile strength decreases significantly. However, at 600 °C, the steel fiber is damaged to a certain extent but not enough to degrade its crack resistance and tensile strength seriously. In addition, due to the internal curing, the deterioration of the interface between the steel fiber and the matrix is also alleviated to a certain extent, so the tensile strength’s decline is slowed down.

(2) Uniaxial tensile stress-strain curve

**Figure 6:**
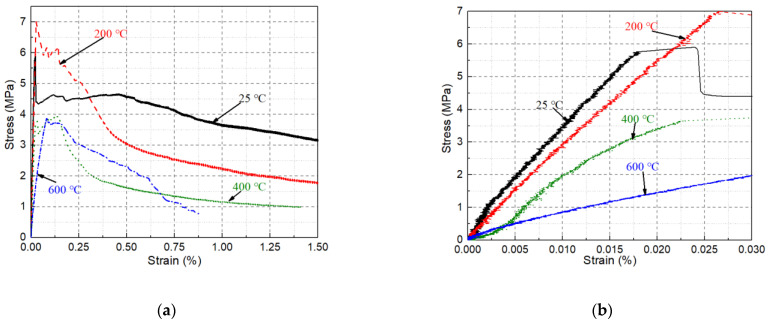
Uniaxial tensile stress-strain curve of heated MSFRC at (**a**) 0–1.5% and (**b**) 0–0.03%.

Figure 6a shows the uniaxial tensile stress-strain curve of MSFRC after high temperature treatment. The stress-strain curves without high temperature treatment at normal temperatures show excellent deformation and load-holding capacities. As shown in Figure 6b, the strain corresponding to the ultimate stress is still relatively small. Also, the load-holding capacity and energy dissipation capacity (the envelope area under the curve) is good. Specifically, at 0.5% and 1.5% strains, the specimen’s stress can remain at more than 75% and 50% of its ultimate stress, respectively. At 200 °C, the specimen’s peak tensile stress is significantly increased, which is mainly due to the internal curing and high temperature drying in the specimen, which improves the strength of the matrix. However, due to the thermal hardening of PVA fibers, the specimens’ deformability and load-holding capacity are reduced [39]. At strain levels of 0.25%, 0.5%, and 1.5%, the specimen’s stress drops to about 70%, 43%, and 25% of its ultimate stress, respectively.

At 400 °C, the specimen’s peak tensile stress significantly decreased, while the strain corresponding to the peak load increased, as shown in Figure 5b. This is mainly due to the deterioration of the matrix at high temperatures and the melting of the PVA fiber, the constraint effect of the PVA fiber on the steel fiber pullout disappears. Simultaneously, the steel fiber and matrix interface also deteriorate, so the steel fiber becomes easier to pull out from the matrix. But this also greatly reduces its peak stress, load holding capacity, and energy dissipation capacity. At strains of 0.25%, 0.5%, and 1.5%, the stress of the specimen drops to about 62.5%, 37.5% and 25% of its ultimate stress, respectively. Nevertheless, the ultimate tensile stress, ultimate strain, and load-holding capacity of specimens at 600 °C do not decrease significantly compared with those at 400 °C. This is mainly due to the good high temperature resistance of the steel fiber and the internal curing and CW phase transformation of the specimen, which improved the specimen’s microstructure. In fact, within the strain range of about 0.2–0.6%, the specimen’s tensile stress value is even higher than that of the specimen treated at 400 °C. In addition, similar to the flexural properties, the uniaxial tensile stiffness of MSFRC under high temperatures deteriorates faster than that of the uniaxial tensile. As shown in Figure 6b, after a 600 °C high temperature treatment, the uniaxial tensile stiffness is reduced to less than 25% of that at room temperature.

### 4.3. Calculation of Uniaxial Tensile and Flexural Strength

(1) Empirical calculation of flexural strength

**Figure 7:**
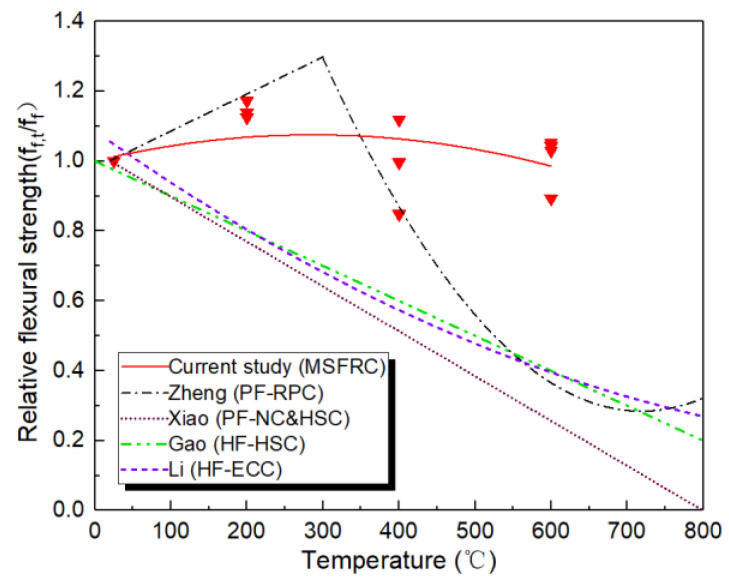
Computation model of flexural strength of heated MSFRC.

For the convenience of engineering applications, following the study of RPC’s mechanical properties after high temperature by Li et al. [43], the relationship between MSFRC flexural strength degrading with high temperature is given, using the quadratic polynomial, as shown in Equation (1) and Figure 7. The correlation coefficient was 0.99, indicating a reasonable degree of correlation.

(1) $$f_{f,t}/f_{f,25\;{}^{\circ}C}=1.02+5.25(t/1000)-9.15{\left(t/1000\right)}^{2}$$

In addition, few studies have been on the high temperature flexural strength of steel-PVA hybrid fiber reinforced cement-based material. Therefore, the calculation models of high-temperature flexural strength of polypropylene fiber reinforced concrete (PF-NC) given by Xiao et al. [44], steel-polypropylene hybrid fiber reinforced concrete (HF-HSC) provided by Gao et al. [45], and polypropylene fiber reactive powder concrete (PF-RPC) given by Zheng et al. [46] are presented in Figure 7 for comparison. At 400 °C, the relative strength of PF-RPC is slightly higher than that of MSFRC herein. This is mainly because the water-binder ratio used in this paper is higher, while RPC’s more compact matrix leads to a more adequate internal curing effect. So the relative flexural strength of PF-RPC after high temperature treatment is improved more obviously. When the temperature reaches 400 °C, the relative flexural strength of MSFRC begins to be better than that of PF-RPC. In addition, the relative flexural strength of MSFRC after the high temperature is always significantly better than that of normal fiber reinforced concrete and high strength concrete. Obviously, the MSFRC designed in this paper achieves the anticipated effect. The CW-PVA-steel multi-scale hybrid fiber system significantly improves the relative bending tensile strength of cement-based materials after high temperatures.

In the literature, only Li et al. [39] reported the high temperature flexural strength of steel-PVA hybrid fiber reinforced cement-based composites (HF-ECC). The test results were also analyzed by a quadratic function, as shown in Equation (2) and Figure 7. The correlation coefficient of Equation (2) can also reach 0.89, indicating a good correlation. It is proven that the quadratic function formula has good adaptability to the high temperature flexural strength of steel-PVA hybrid fiber reinforced cement-based composites with lower steel fiber content. It is shown in Figure 7 that due to the low content of steel fiber and high content of PVA in HF-ECC, the relative flexural strength begins to decrease significantly after the PVA fiber is melted below 300 °C. Meanwhile, the relative flexural strength of MSFRC is higher than that of HF-ECC due to its higher steel fiber content.

(2) $$f_{f,t}/f_{f,25\;{}^{\circ}C}=0.92-0.39(t/1000)-0.31{\left(t/1000\right)}^{2}$$

(2) Empirical calculation of uniaxial tensile strength

**Figure 8:**
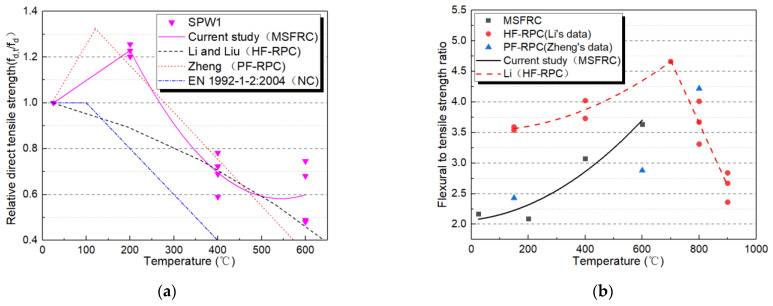
Computation model of uniaxial tensile strength of heated MSFRC (**a**) Relative uniaxial tensile strength versus temperature; (**b**) Flexural to uniaxial tensile strength ratio versus temperature.

For the convenience of engineering application, concerning the study of RPC after high temperature by Li and Liu [43], the relationship formula of uniaxial tensile strength of MSFRC degrading with high temperature was adopted in a segmented form, as shown in Equation (3) and Figure 8a. In Equation (3), the correlation coefficients of linear and quadratic polynomial segments are 0.95 and 0.89, respectively, which show a good correlation.

(3) $$f_{d,t}/f_{d,25\;{}^{\circ}C}=\{\begin{array}{c} 0.97+1.31(t/1000),25\;{}^{\circ}C\leq t\leq 200\;{}^{\circ}C\\ 2.20-5.93(t/1000)+5.44{\left(t/1000\right)}^{2},200\;{}^{\circ}C\leq t\leq 600\;{}^{\circ}C \end{array}$$

In addition, the calculation model of uniaxial tensile strength of steel-polypropylene HF-RPC given by Li and Liu [43], PF-RPC is provided by Zheng et al. [46], and the normal concrete (NC) model recommended by European code EN 1992-1-2: 2004 [47] are also illustrated in Figure 8a. In general, the variation trend of PF-RPC’s relative uniaxial tensile strength after the high temperature is consistent with the results of MSFRC. The uniaxial tensile strength of PF-RPC was slightly higher than that of MSFRC when the temperature was within 200 °C. When the temperature reaches 500 °C, the relative uniaxial tensile strength of MSFRC is higher than that of PF-RPC. The absolute value of tensile strength and energy dissipation capacity of PF-RPC is significantly lower than those of MSFRC because no steel fiber is used. Compared with HF-RPC test results, it can be seen that the deterioration rate of MSFRC uniaxial tensile strength with temperature is significantly slower than that of HF-RPC. The deterioration rate of the uniaxial tensile strength of all fiber reinforced cement-based materials is slower than that of NC. Obviously, the MSFRC designed in this paper has achieved the expected effect, and the CW-PVA-steel multi-scale hybrid fiber system has significantly improved the deterioration of the uniaxial tensile strength of cement-based materials under high temperatures.

In addition, the relationship between the flexural strength and the uniaxial tensile strength ratio of MSFRC at high temperatures is presented. The relationship is a quadratic function, as shown in Equation (4) and Figure 8b. Equation (4)’s correlation coefficient is 0.80, indicating a reasonable degree of correlation. Figure 8b simultaneously plots the high-temperature flexural to uniaxial tensile strength ratio of HF-RPC developed by Li and Liu [43] and PF-RPC by Zheng et al. [46] as a reference. As shown in Equation (5), the correlation coefficient of quadratic polynomial and straight line segments are 0.90 and 0.89, respectively, which is in good agreement. In general, the flexural to uniaxial tensile strength ratio of steel-PP HF-RPC at high temperature is higher than that of MSFRC in this paper.

(4) $$f_{f,t}/f_{d,t}=2.07+0.50(t/1000)+3.70{\left(t/1000\right)}^{2},25\;{}^{\circ}C\leq t\leq 700\;{}^{\circ}C$$

(5) $$f_{f,t}/f_{d,t}=\{\begin{array}{c} 3.53-0.14(t/1000)+2.50{\left(t/1000\right)}^{2},25\;{}^{\circ}C\leq t\leq 700\;{}^{\circ}C\\ 11.84-10.24(t/1000),700\;{}^{\circ}C\leq t\leq 900\;{}^{\circ}C \end{array}$$

The relationship between flexural and uniaxial tensile strength can be described as a linear formula shown in Equation (6), with a correlation coefficient of 0.60.

(6) $$f_{d,t}=1.3126f_{f,t}-11.815$$

## 5. Microscopic Morphology Observation

**Figure 9:**
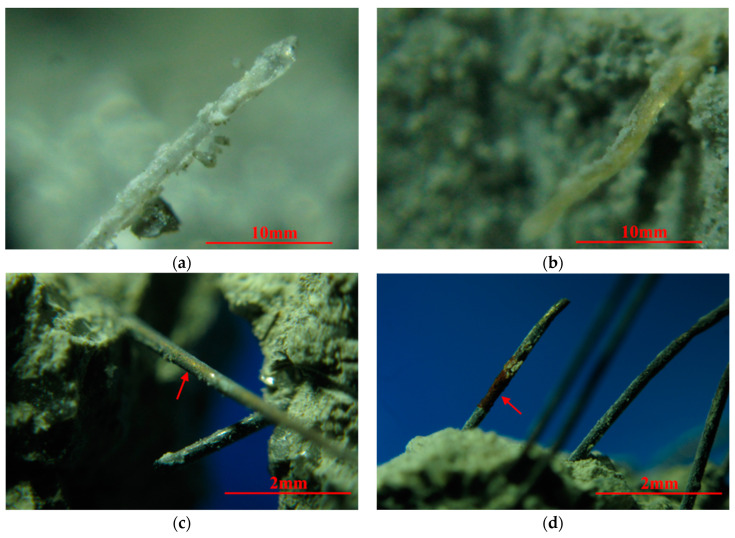
MSFRC at (**a**) Room temperature, (**b**) 200 °C, (**c**) 400 °C and (**d**) 600 °C under optical microscope.

After crushing MSFRC specimens under applied loading, the fragments were collected for observation by an optical microscope. Large amounts of cement paste, and even large chunks of mortar, were attached to the PVA fiber surface at room temperature, as shown in Figure 9a. This indicates that the PVA fiber is bonded closely to the matrix and has a good cooperative working performance. The reason is that the abundant hydroxyl groups on the PVA fiber surface lead to its strong hydrophilicity, thus enhancing the bonding force between the PVA fiber and the matrix. However, after 200 °C, the colorless and transparent PVA fibers became light yellow, as shown in Figure 9b. The reason is that in an alkaline environment, PVA fibers undergo an oxidation reaction and elimination reaction after high temperature treatment, resulting in changes in the chemical composition of PVA fibers [39]. Double bond C=O was generated when exposed to heat, resulting in higher rigidity of the main molecular chain. Therefore, the tensile strength of PVA fiber was increased, and PVA fiber’s elongation decreased at 200 °C [39]. As a result, the strength of MSFRC increased after high temperature treatment at 200 °C, while the deformation capacity decreased. The results of flexural properties and uniaxial tensile properties in Section 4.1 and Section 4.2 confirmed the above analysis. The change of uniaxial tensile properties was more evident than that of bending tensile properties.

The arrow in Figure 9c shows the steel fiber bridging cracks in the mortar at 400 °C. The yellow copper coating can still be observed on the steel fiber’s surface, which proves that the steel fiber has not been significantly oxidized at 400 °C. However, PVA fibers could not be observed in MSFRC at 400 °C, as shown in Figure 9c. The melting of PVA fiber will degrade the deformation ability of MSFRC to a certain extent, but the holes left after melting can release steam pressure in the fire’s high temperature and avoid the high temperature burst of cement concrete. However, at 600 °C, the steel fibers in cement-based materials oxidized significantly, and the local color changed to a brown similar to that of rust. The cross-section size of steel fibers decreased under high temperature oxidation, as shown by the arrow in Figure 9d. Comparing the results of Section 4.1 and Section 4.2, the degradation of steel fiber and PVA fiber at 400–600 °C significantly deteriorates the uniaxial tensile properties of MSFRC. Still, the flexural properties’ degradation is not obvious, which is related to the difference between the specimen size and stress state.

**Figure 10:**
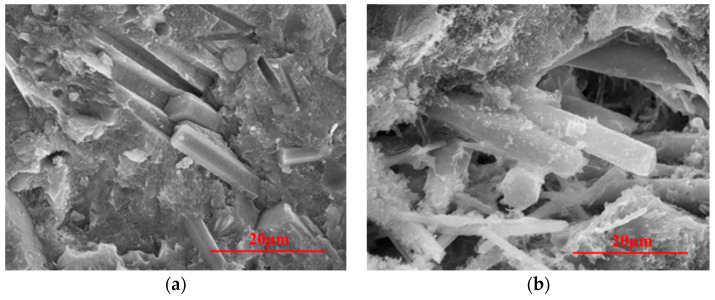
MSFRC at (**a**) Room temperature and (**b**) 600 °C under SEM.

The micron scale CW is challenging to identify under the optical microscope in Figure 9. Under SEM in Figure 10, CW dispersion in the matrix is shown. In Figure 10a, the pore channels left after CW pullout and tensile fracture can be seen at room temperature, verifying the effectiveness of CW in crack inhibition enhancement at the microscopic level. At 600 °C, CW’s overall morphology and structure generally remain intact, and there is no significant deterioration compared with that at room temperature [33,35]. Figure 10b shows the morphology of a defect filled and bridged by CW. Figure 10b also indicates that hydration products on the CW surface at 600 °C are significantly more common than those at room temperature. This shows that new hydration products are generated on CW’s surface, which confirms that the synergistic effect of “internal curing” of hot steam and CW ultimately resulted in improved mechanical properties of MSFRC at high temperatures.

## 6. Conclusions

In this paper, the uniaxial tensile and flexural properties of multi-scale fiber reinforced cement-based materials (MSFRC) at high temperatures and the related empirical calculation methods are studied. The conclusions are as follows:

(1) The residual flexural and uniaxial tensile strength and toughness of MSFRC decreased with increased temperatures but decreased slowly at 600 °C and increased slightly at 200 °C. The deformation capacity of MSFRC (peak flexural deflection and peak tensile strain) decreased first and then improved with the temperature increase.
(2) The nominal stiffness of MSFRC under flexural and uniaxial tensile loads decreased gradually with the increased temperature. The decrease rate of stiffness was more rapid than that of the strength, and the curve became flat gradually. However, the flexural properties are different from the uniaxial tensile properties. The flexural deflection curve deteriorates slowly or even improves slightly at 500 °C. The tensile properties of MSFRC are more sensitive to temperature changes. The load bearing capacity of MSFRC decreased by more than 30% at 200 °C, dropping sharply by about 70% at 400 °C.
(3) Based on the above experimental results, the prediction models for the quantitative relationship between residual flexural (uniaxial tensile) strength and temperature of MSFRC was established. The calculation results of the models were close to the experimental results. Compared with the existing research results and prediction models in the literature, the new MSFRC has achieved an excellent high temperature resistance. The CW-PVA-steel multi-scale hybrid fiber system significantly alleviates the deterioration of mechanical properties of cement-based materials after high temperature, and improves the mechanical properties of cement-based composites at high temperatures.
(4) The multi-scale morphology observation of OM and SEM shows that the flexural and uniaxial tensile properties of MSFRC at high temperatures result from the physical and chemical changes of steel fiber, PVA fiber, and calcium carbonate whisker at different temperatures.

## Figures and Tables

**Table 1:** Properties of raw materials.

Raw Materials	Density (g/cm^3^)	Size	Mechanical Property	Origin
Cement	3.20	Specific surface area 356 m^2^/kg	-	Dalian Onoda Cement Co. Ltd.
Silica sand	2.65	Fineness modulus 1.9 Media sand	Moh’s hardness 7	Dalian
Steel fiber	7.8	Length 13 mm Diameter 200 μm	Tensile strength ≥ 2 GPa Elastic modulus 200–210 GPa	Bekaert
PVA fiber	1.29	Length 6 mm Diameter 31 μm	Tensile strength 1.1 GPa Elastic modulus 41 GPa	Wanwei High-tech Material Co. Ltd., Hefei, China
CaCO_3_ whisker	2.86	Length 20–30 μm Diameter 0.5–2 μm	Tensile strength 3–6 GPa Elastic modulus 410–710 GPa	Shanghai Fengzhu Co. Ltd.

## Data Availability

Data are available on request from the authors.

## Nomenclature

CW	CaCO_3_ whisker
PVA	polyvinyl alcohol
MSFRC	multi-scale fiber reinforced cement matrix composites
ECC	engineered cement-based composites
OM	optical microscope
SEM	scanning electron microscope
NC	normal concrete
PF-NC	polypropylene fiber reinforced normal concrete
HF-HSC	hybrid fiber reinforced high strength concrete
PF-RPC	polypropylene fiber reinforced reactive powder concrete
HF-ECC	hybrid fiber reinforced cement-based composites
